# Circular RNA in Diseased Heart

**DOI:** 10.3390/cells9051240

**Published:** 2020-05-17

**Authors:** Ying Wang, Bin Liu

**Affiliations:** Department of Biological Sciences, Mississippi State University, Mississippi State, MS 39762, USA

**Keywords:** circRNA, ciRNA, microRNA sponge, protein sponge, hypertrophy, dilation, infarction, atrial fibrillation, biomarker

## Abstract

Heart disease remains the leading cause of death globally and leads to tremendous socio-economic burden. Despite advances in the field of cardiovascular research, novel theranostics are still in urgent need. Remarkable progress has been made in understanding aberrant protein interactions and signaling pathways in the diseased heart, but less is known regarding epigenetic regulation. Non-coding RNAs have emerged as important regulators of cardiac function and have been implicated in disease. While significant progress has been made in understanding the roles of microRNAs and long non-coding RNAs, the functional roles of circular RNAs are less explored. Recent studies have provided ample evidence supporting their roles in multiple physiological processes including regulating the function of the heart. Compared with other RNAs, circular RNAs exhibit higher stability and more versatile functional modes: including sponging microRNAs, scaffolding proteins, regulating transcription, and even encoding small regulatory peptides. These characteristics make circular RNAs promising candidates for the development of diagnostic tools and therapies for heart disease. In this review, we will discuss the biogenesis of circular RNAs and provide an update of their functional implications in heart disease, with an emphasis on heart failure and arrhythmias.

## 1. Introduction

Most RNAs exist in the linear form as a direct result of the transcription. However, post-transcriptional processing leads to the generation of various groups of circular RNAs. The circular genomes of four viroids are the first circular RNAs identified in nature [1], followed by other viroids and a human subviral agent, the hepatitis delta virus [2]. The existence of cellular RNAs in the circular form has been implied as early as in 1979 [3]. Particularly, circular RNAs generated from precursor mRNAs through backsplicing (circRNAs) or from splicing lariats that escaped from debranching (ciRNAs) have become a new research front across disciplines [4,5,6,7]. Henceforth, we will use circular RNAs to refer to circRNAs and ciRNAs. Most of such circular RNAs express at low levels and are not conserved during evolution [4,5], resembling less conserved microRNAs [8,9,10]. Nevertheless, some have been found to accumulate at high levels in a particular species, a particular organ or a particular biological process, likely attributable to the selection pressure [4,5]. Despite the lack of functional annotations for most of the circular RNAs, functional studies on a few circular RNAs already demonstrated that circular RNAs can be potent regulators of gene expression [4,5,7].

Heart disease remains the leading cause of death worldwide, thus the development of novel theranostics is still required. As a muscular pump, the primary function of the heart is to generate sufficient force to drive blood through circulation. Heart failure (HF) is a condition in which the heart cannot perform as an efficient pump, thus threatening patients’ lives. Causes for HF include coronary heart disease, hypertension, valvular disease and genetic cardiomyopathies. Sometimes occurring concurrently with HF, cardiac arrhythmia is a disorder of heart rhythm that share a certain group of etiologies with HF. Of note, the cardiac arrhythmia by itself, in particular ventricular arrhythmia, could lead to sudden cardiac death [11]. With growing evidence of non-coding RNA participating in heart disease progression, they have emerged as promising targets for diagnosis and treatment. Although substantial progress has been made in understanding the functional roles of microRNAs and long non-coding RNAs in heart disease, far less is known about the roles of circular RNAs. In this review, we will introduce known mechanisms of circular RNAs biogenesis and functional modes in general, as well as their implications in heart failure and arrhythmias.

## 2. Biogenesis of Circular RNAs

With the rapid development of various genome-wide sequencing tools, an explosive number of circular RNAs have been identified. In contrast to conventional RNA-Seq procedures, RNA samples need special treatments to enrich circular RNAs before subject to sequencing. A combination of ribosomal RNA depletion (ribo-depletion) and mRNA removal (poly A-depletion) can remove the most abundant ribosomal RNAs and mRNAs thus enrich circular RNAs. Alternatively, RNase R treatment, which only digests linear RNAs, can also enrich circular RNAs. The treated samples can then be sequenced for identification of circular RNAs [4,5,6].

**Figure 1:**
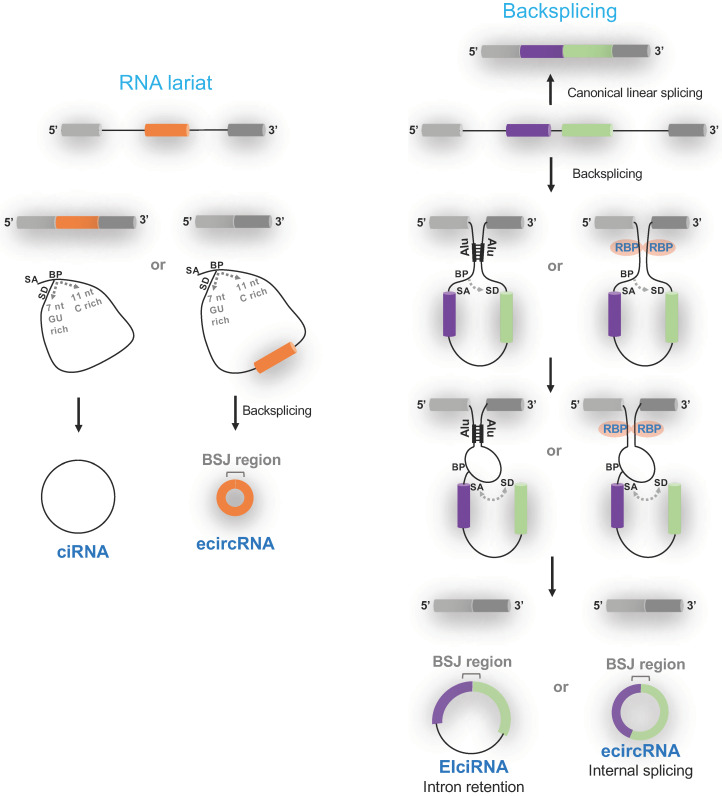
Biogenesis of circular RNAs. A signature, 7-nt GU rich motif near the 5′ splice site and an 11-nt C-rich motif at the branchpoint site, is critical for RNA lariats to escape debranching, thereby generating ciRNAs and ecircRNAs (after another backsplicing step). Backsplicing occurs in the presence of flanking inverted repeat elements (e.g., Alu elements) and/or with the aid of RNA-binding proteins (RBP). EIciRNAs and ecircRNA are both circRNAs, which require backsplicing in biogenesis. SA, splicing acceptor. SD, splicing donor. BP, branching point. BSJ, backsplice joint. Colored boxes depict exons. Black lines depict introns.

To date, circular RNAs can be categorized as circular intronic RNAs (ciRNAs) [12], exon-intron circRNAs (EIciRNAs) [13] and Exonic circRNAs (ecircRNA) based on the splicing mechanism and the presence of exons/introns (Figure 1) [4,5,14,15,16]. It is noteworthy that ciRNAs and circRNAs have distinct chemical features- a 2′-5′ carbon linkage only existing at the splicing branch-point in ciRNAs [12]. During mRNA processing, introns are spliced out in the lariat form, which is normally subject to debranching to become linear RNAs for turnover [17]. Some intron lariats, specifically those including a signature of 7-nt GU-rich motif near the 5′ splice site and an 11-nt C-rich motif at the branchpoint site, escape debranching and turnover to become ciRNAs (Figure 1) [12]. The mechanism for the transition from RNA lariats to circular RNAs remains unknown. In the case of RNA lariats possessing the signature and originated from exon skipping, an additional backsplicing will occur to remove intron sequences as well as the 2′-5′ carbon linkage, resulting in ecircRNAs (Figure 1) [13]. Although the introns in these exon-containing RNA lariats may be retained to generate EIciRNAs in theory [18,19], such an example awaits to be identified.

Besides originated from RNA lariats, backsplicing is the other major mechanism in generating circRNAs [20,21]. Unlike the canonical splicing where a splice donor site resides in the upstream of a splice acceptor site, some splice donor sites interact with their upstream splice acceptor sites instead, forming a so-called backsplicing that is not in the canonical 5′ to 3′ direction (Figure 1). Inverted repeat elements (*cis*-elements such as Alu elements) flanking donor and acceptor splice sites [22,23,24,25] often form base pairings to promote backsplicing (Figure 1). Alternatively, RNA binding proteins interact with specific motifs flanking donor and splice sites to promote backsplicing (Figure 1) [26,27,28,29,30,31,32]. Following the formation of circular RNAs by backsplicing, further splicing can occur to remove internal introns, resulting in ecircRNAs or EIciRNAs (in the case of intron retention). Splicing within circRNAs is subject to the regulation of all four possible alternative splicing modes (i.e., exon skipping, intron retention, alternative 5′ splicing and alternative 3′ splicing), thus greatly diversifying circRNA species in general [33].

The production of some circular RNAs may be specifically regulated in an organ-specific manner. Despite that most circular RNAs are generated from constitutive exons in hearts, a critical splicing factor involved in dilated cardiomyopathy (i.e., RBM20) specifically regulates the generation of ecircRNAs from skipped exons corresponding to the I-band region of the Titin gene [27,34]. Since this subset of ecircRNAs is mainly generated from skipped exons, in other words alternatively spliced introns, they represent the unique biogenesis of ecircRNAs from RNA lariats instead of the canonical backsplicing pathway. Interestingly, RBM20 is among the most frequently affected genes in dilated cardiomyopathy [35]. In addition, both RBM20 and these Titin-derived circRNAs seem to regulate the progression of dilated cardiomyopathy, highlighting the importance of organ-specific biogenesis and function of circRNAs.

## 3. Functional Modes of Circular RNAs

Numerous molecular and biochemical tools have been developed to study the functions of ciRNAs and circRNAs. ciRNAs and circRNAs can be ectopically expressed using specially designed vectors or silenced by using siRNA or antisense morpholino [5,6,7]. They can also be deleted by using CRISPR/Cas9 [5,6,7]. Protein-centric or RNA-centric purification schemes can help identify the interacting partners of circRNAs and ciRNAs [4], thus unraveling their functional mechanisms. Indeed, aided by these tools, substantial progress has been made in the past decade, demonstrating that circRNAs and ciRNAs can exert diverse functions.

**Figure 2:**
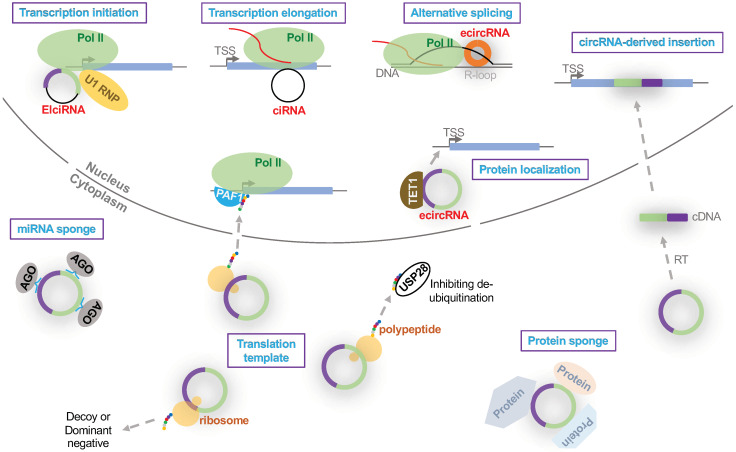
**Potential functional modes of circular RNAs.** The biological processes regulated by circular RNAs are highlighted in purple boxes. Red lines depict nascent RNA transcripts. TSS, transcription starting site. Pol II, RNA polymerase II. PAF1, RNA polymerase II-associated factor 1 homolog. AGO, Argonaut proteins. TET1, Tet methylcytosine dioxygenase 1. USP28, ubiquitin carboxyl-terminal hydrolase 28. RT, reverse transcription. miRNA, microRNA.

The vast majority of circRNAs, particularly ecircRNAs, dominantly accumulate in cytoplasm and exert diverse regulatory roles [4,5,7,36]. ecircRNAs are the most studied group of circular RNAs. Some of ecircRNAs have been demonstrated to serve as sponges to sequester microRNAs [37,38,39,40,41,42,43,44,45] or protein factors [46,47,48,49]. These two functional modes are also well-characterized in the diseased heart. When circRNAs serve as sponges to sequester certain microRNAs via sequence complementation, they prevent those sequestered microRNAs from targeting cellular mRNAs (Figure 2). The molar ratio between circRNAs and microRNAs in cells is thus critical for the sponge effect because high concentration of circRNAs increases their opportunities to sequester microRNAs. It is notable that this mode can also be used to degrade certain circRNAs. For instance, miR-671 can guide the cleavage of a conserved circRNA *CDR1as* [50]. To exert sponge activity or to be degraded is probably determined by the extent of base pairings between microRNA and circRNA, as the perfect complementation often leads to cleavage [50]. The examples of ecircRNAs functioning as microRNA sponges in various heart diseases are discussed in details below.

circRNA-protein interactions in cytoplasm can exert diverse functions (Figure 2). circRNAs can act as a sponge to sequester cellular proteins [46,47,48,49]. For instance, *circFOXO3* is highly expressed in mammalian hearts and interacts with ID-1, E2F1, FAK and HIF1α to promote cardiac senescence [46]. More details regarding the function of *circFOXO3* in the diseased heart are discussed below. Alternatively, particular proteins can be activated to bind various circRNAs. For instance, antiviral protein NF90/NF110 and protein kinase R can bind various cytoplasmic circRNAs upon viral infections to elicit prompt immune responses [28,51].

Recently, a small sub-set of endogenous circRNAs (i.e., *circ-FBXW7*, *circMB1*, *circPINTrxon2*, *circ-SHPRH* and *circ-ZNF609*) have been demonstrated as translatable by generating polypeptides [52,53,54,55,56,57] (Figure 2), despite the lack of both the m^7^G caps and the poly A tails. As a reservoir for future explorations, thousands of circRNAs are predicted to include a putative open reading frame with an upstream IRES (internal ribosomal entry site) [58]. The function of circRNA-derived polypeptides is beginning to be elucidated recently. FBXW-185aa, a polypeptide derived from *circ-FBXW7*, interacts with a de-ubiquitinating enzyme USP28 to release the inhibition on FBXW7α-induced degradation of an oncoprotein MYC [55]. PINT87aa, a polypeptide derived from *circPINTrxon2*, interacts with Pol II-associated factor 1 (PAF1) to tightly interact with target gene promoters and decrease Pol II elongation efficiency. As a consequence, the expression of certain oncogenes, such as CPEB1, SOX-1 and MYC, is reduced [57]. SHPRH-146aa, a polypeptide derived from *circ-SHPRH*, may serve as a decoy to protect the full-length SHPRH protein from ubiquitin-based degradation [56].

ecircRNA can also exert functions in the nucleus. For instance, a circRNA derived from the FLI1 gene can recruit TET1 to specific gene promoters to regulate gene expression (Figure 2), as a novel mode of guiding protein subcellular localization [59]. In another recent example, a plant circRNA derived from the SEP3 gene can interact with the cognate gene region in the chromosome to form an R-loop (DNA–RNA hybrid), which halts transcription and results in alternative splicing (Figure 2) [60]. It is unclear whether there is any nucleocytoplasmic shuttling of those ecircRNAs functioning in the nucleus. If such a shuttling process is detected, it would be interesting to further understand the nuclear import regulation because very few RNAs are selected for nuclear import. Otherwise, it remains an intriguing question of how particular ecircRNAs are retained in the nucleus.

A recent report shows that a few circRNAs can serve as templates for reverse transcription, followed by integration into genome resulting in circRNA-derived pseudogenes (Figure 2). Such genome insertion can be disruptive for gene expression if occurring in the gene body or the regulatory regions, as exemplified by *circSATB1*-derived pseudogene locus in mouse [61].

In contrast to ecircRNAs, EIciRNAs and ciRNAs are less studied. In particular, ciRNAs are often overlooked when samples are not treated to remove the poly A-containing mRNAs [12]. EIciRNAs and ciRNAs are dominantly localized in the nucleus [12,13]. A human ciRNA, *ci-ankrd52*, accumulates at the site of transcription and associates with Pol II elongation machinery to play a positive regulatory role in *cis* (Figure 2) [12]. Similarly, several human EIciRNAs have been shown to interact with U1 snRNP and Pol II to promote the expression of their parental genes in *cis* [13]. The EIciRNA-U1 snRNP-Pol II complexes appear to modulate gene expression through interacting with gene promoter regions (Figure 2).

## 4. Expression Atlas of Circular RNAs in Heart Failure

Several studies examined the circRNA expression profile in HF-related models, using samples from rodents and/or humans. Werfel et al. characterized circRNA expression in human, mouse and rat hearts and identified >9000 candidate circRNAs for each species [62]. Among them, only about 30% were conserved between mouse and rat, and about 10% were conserved across all three species. Rather than being regulated by cardiac disease (human HF samples), the expression profile of circRNAs appears to be regulated more strongly by developmental stages (neonatal vs adult). This finding is also supported by an independent profiling study by Tan et al. which reported a lack of differential expression of circRNAs in diseased hearts when comparing HF human samples or transverse-aortic constriction (TAC) mice samples to their respective controls [63]. The most abundant circRNA identified in this study is *circSLC8A1-1*, whose parental gene encodes the sodium-calcium exchanger (NCX). Other highly expressed circRNAs correspond to genes encoding Titin, ryanodine receptor 2 and Dystrophin. The lack of disease-related differential expression in circRNAs was ascribed to the higher stability of circRNAs as compared with linear forms of RNAs such as mRNA [22]. It is also hypothesized that although its expression profile remains unchanged, its interaction with microRNA and subsequent regulations is affected by disease states [63].

In a study on human hypertrophic and dilated cardiomyopathy (HCM and DCM), 60 and 43 differentially expressed circRNAs were identified via RNA-Seq in HCM and DCM patients, respectively when compared with healthy donors [27]. Among the 826 circRNAs uncovered from all three groups, 80 circRNAs are expressed from the Titin gene, which is known to undergo complex alternative splicing [64]. Some of these *Titin*-derived circRNAs appear to be regulated in DCM, but not HCM [27]. When examined in a DCM model due to the ablation of RBM20, a splicing factor known to regulate alternative splicing of *Titin*, a specific subset of circRNAs originated from RBM20-regulated I-band region of *Titin* transcript are lost [27]. These results point to the possibility that *Titin*-derived circRNAs are involved in DCM, which is supported by an independent study showing that inhibition of *Titin*-derived circRNAs increased the susceptibility of cardiomyocytes to doxorubicin cardiotoxicity [65], a condition manifested as a DCM-like phenotype in vivo. Although this study seems to report more differentially expressed circRNAs under disease conditions, its conclusion is based on a limited sample size (n = 2 for each group) [27]. But still, a recent profiling study of RNA-Seq reported 303 upregulated and 98 downregulated circRNAs in isoproterenol (ISO)-induced hypertrophy mouse model [66]. Thus, it remains inconclusive whether heart diseases alter the circRNA expression profile extensively.

## 5. Circular RNA Functions in Heart Failure

### 5.1. HF Due to Non-Ischemic Cardiomyopathy: Hypertrophy

HRCR is the first identified circRNA with a functional role in cardiac hypertrophy [67]. The expression of HRCR was downregulated in mouse models of hypertrophy induced by ISO or TAC, while the expression of miR-223* (termed miR-223-5p in the reference) was upregulated. The authors show that HRCR interacts with miR-223* in vivo to regulate the expression level of Apoptosis repressor with CARD domain (ARC) to mediate hypertrophy. Viral-mediated overexpression of HRCR attenuated hypertrophy by preserving the level of ARC. It is notable that both miR-223 and miR-223* are involved in suppressing necroptosis in ischemic/reperfused hearts through regulating their own targets [68]. Future analyses on reported miR-223* targets other than ARC as well as the impact of HRCR on the function of miR223 may provide new mechanistic insight into this process.

Although detected as unaltered by disease conditions in mouse, *circSLC8A1-1*, a highly expressed circRNA, was found to play a role in hypertrophy by sponging miR-133, a well-recognized regulator of cardiac hypertrophy [69]. While adeno-associated virus serotype 9 (AAV9)-mediated repression of *circSLC8A1-1* expression alleviated hypertrophy in a pressure-overload model; overexpression of *circSLC8A1-1* led to HF. Of note, the expression of *circSLC8A1-1* was shown upregulated in human DCM patients along with 3 other differentially expressed circRNAs elsewhere [70]. Additionally, *circSLC8A1-1* was also reported to be upregulated in ischemic rat cardiac cells and mouse heart, and mediate ischemic myocardial injury (see below) [71]. Despite that the expression level of *circSLC8A1-1* varies in diseased hearts from different species/models, all these studies support the notion that highly expressed, conserved circRNAs are more likely to play a functional role.

A recent study by Li et al. reported the role of *circRNA_000203* in Ang-II induced hypertrophy [72]. In this study, the upregulation of this circRNA was confirmed in both myocardium of Ang-II infused mice and a cellular model of Ang-II treated neonatal mouse ventricular cardiomyocytes (NMVC). Overexpression of *circRNA_000203* induced cellular hypertrophy in NMVC and exacerbated Ang-II induced hypertrophy in a transgenic mouse model in vivo (TG-circ203). Mechanistically, *circRNA_000203* sponges miR-26b-5p and miR-140-3p, abolished their suppression of Gata4, a hypertrophy-responsive transcription factor. Additionally, upregulation of *circRNA_000203* in Ang-II induced hypertrophy was due to activation of NF-KB signaling pathway.

### 5.2. HF Due to Non-Ischemic Cardiomyopathy: Dilation

Doxorubicin (DOX), a chemotherapeutic agent induces chronic cardiotoxicity, which is characterized by morphologic and functional derangements similar to DCM [73]. In two independent studies from the same group, two circRNAs are identified as differentially expressed and playing functional roles when comparing young and old hearts [46,47]. *circFOXO3* is highly expressed in aged human and mice hearts, along with markers of cellular senescence [46]. Overexpression and downregulation of *circFOXO3* exacerbated and alleviated DOX-induced cardiomyopathy, respectively. Cellular senescence was also exacerbated by *circFOXO3* overexpression but inhibited by its silencing. Mechanistically, *circFOXO3* binds anti-senescent and anti-stress protein factors (ID-1, E2F1, FAK and H1F1α) and retains them in cytoplasm to repress their beneficial activity thereby promoting senescence [46]. In contrast, *circAmotl1* is highly expressed in neonatal human heart tissue and promote cardiomyocyte survival [47]. Its expression protected against DOX-induced cardiomyopathy in mouse. Further, *circAmotl1* binds to and activates phosphorylation and nuclear translocation of AKT, a cardio-protective molecule, thus enhancing cardiac repair [47].

More evidence supporting the role of circRNAs in dilated cardiomyopathy is provided by a study on the RNA-binding protein Quaking (Qki) [65]. AAV9-mediated overexpression of Qki5 prevented cardiac apoptosis and dilation in DOX-induced cardiomyopathy. The protective effect of Qki5 in DOX-induced cardiomyopathy works through regulating the expression of a specific group of circRNAs, including those derived from Titin, Fhod3 and Strn3 [65]. In particular, inhibition of *Titin*-derived circRNAs increased the susceptibility of cardiac cell lines to DOX toxicity [65]. These results are also consistent with a previous study that highlighted the role of *Titin*-derived circRNAs in DCM [27]. Additionally, a recent study on DOX cardiotoxicity revealed the role of circRNA *Pan3*, which is negatively regulated by miR-31-5p by directly targeting *Qki*, thus inducing apoptosis [74].

### 5.3. HF Due to Ischemic Heart Disease

A circRNA, *CDR1as*, previously shown to sponge miR-7a in the brain [38], has been shown to play a role in myocardial infarction (MI) [75]. Geng et al. reported the upregulation of both *CDR1as* and miR-7 in MI mice. Overexpression of *CDR1as* promoted cell apoptosis in vitro and increased cardiac infarct size in vivo, which can be reversed by miR-7a overexpression. However, an unconventional in vivo transfection technique was employed, the validity of which needs to be further demonstrated. Another study uncovered the role of a mitochondrial fission and apoptosis-related circRNA (*MFACR*) in MI [76]. *MFACR* sponges miR-652-3p to regulate expression of MTP18, a mitochondria membrane protein related to mitochondrial fission. Virus-mediated in vivo downregulation of *MFACR* attenuated MI in mice.

In addition to its involvement in non-ischemic cardiomyopathy, an NCX-derived circRNA, *circNCX1*, plays a role in MI through a similar mechanism of sponging miR-133a-3p, although with a different target protein of pro-apoptotic gene cell death-inducing protein (CDIP1) [71]. Suppressing the expression of *circNCX1* reduced expression of CDIP1 and attenuated apoptosis and ischemia/reperfusion injury.

A cardioprotective circRNA, *circTtc3*, was found markedly upregulated in ischemic myocardium and cardiomyocytes experiencing hypoxia [77]. AAV9-mediated downregulation of *circTtc3* exacerbated cardiac dysfunction in a rat model of MI. *circTtc3* sponges miR-15b-5p to upregulate the expression of Arl2, which is partially responsible for the beneficial effect of *circTtc3* overexpression in cardiomyocytes.

While several profiling studies provided a pool of circRNAs with altered expression under disease conditions, it is challenging to further identify the essential subgroup that is functionally important. Huang et al. attempted to identify such candidates through analyzing the relationship between super-enhancers and circRNA network. They identified *circNfix* as a key circRNA involved in cardiac regeneration [78]. Downregulation of *circNfix* promoted myocyte proliferation, angiogenesis and attenuated cell death and cardiac dysfunction post MI. Two parallel mechanisms contribute to the functional roles of *circNfix* in myocyte proliferation and cardiac regeneration. *circNfix* promotes the interaction of Ybx1 (a transcription factor related to cell proliferation) and Nedd4l (an E3 ubiquitin ligase), thus inducing degradation of Ybx1 and repressing the expression of cyclin A2 and B1. Additionally, *circNfix* sponges miR-214 to promote Gsk3β expression to inhibit proliferation and angiogenesis via degrading β-catenin and inhibiting the secretion of an angiogenic factor, VEGF, respectively [78].

*circFndc3b* is another circRNA reported to modulate cardiac regeneration post MI [79]. It is downregulated in heart tissue from both post-MI mouse and ischemic cardiomyopathy patients. AAV9-mediated overexpression of *circFndc3b* attenuated apoptosis, improved angiogenesis and contractile function in post-MI mouse models. This is due to the interaction between *circFndc3b* and the RNA binding protein FUS, which regulates the expression of VEGF-A and angiogenesis.

While our discussions mainly focus on the MI models, ischemic heart disease also includes coronary artery disease caused by atherosclerosis. Several circRNAs have been identified as playing roles in coronary artery disease, readers are referred to the original papers or review articles on this topic for details [36,80,81,82].

## 6. Circular RNAs Expression Atlas in Arrhythmia

Atrial Fibrillation (AF) is the most common form of cardiac arrhythmias and associated with significant morbidity and mortality [83]. At least three independent profiling studies based on samples from AF patients have been reported. Among them, two studies identified quite substantial changes in the expression level of circRNA in hearts from AF patients, with ~700 and ~300 differentially expressed circRNAs detected by microarray and RNA-Seq, respectively [84,85]. In contrast, an independent human study only reported 20 upregulated and 3 downregulated circRNAs in the setting of AF, using RNA-Seq [86]. All these studies are limited by the relatively small sample size (from 3 to 5 samples per group). Further, in some of these studies, the age for the control group is either not provided [85] or significantly younger (35–40 yr) than the AF patient group (68–75 yr) [84], which also has existing complications, thus compromising the analysis. Lastly, an independent profiling study on the canine rapid atrial pacing model has identified 106 upregulated and 40 downregulated circRNAs [87]. Thus, no consensus has been reached as to whether AF is associated with extensive alteration of circRNA expression profile.

A recent study raised the idea that circRNAs may function in different phases of the disease in a temporally regulated fashion. It has been predicted in silico that the crosstalk between 107 microRNAs and 9 circRNAs may occur when AF proceeds from paroxysmal to permanent AF [88]. However, this prediction assumes that circRNAs and their sequestered microRNAs display inversely correlated expression, which contradicts many observations that microRNAs and their cognate sponge circRNAs are simultaneously expressed at high levels [37,39,41,43].

## 7. Circular RNAs as Biomarkers in Heart Disease

There have been growing interests in identifying non-coding RNAs as biomarkers of heart disease. While a vast body of literature has reported microRNAs as promising candidates of biomarkers in multiple cardiovascular diseases [89], the role of circRNAs is less recognized. Compared with the linear form of RNA, circular RNAs are generally thought to possess higher stability due to the lack of free ends, thus rendering them resistant to exonuclease-mediated degradation [22]. However, it is also reported that circRNAs may only briefly exist in serum with a half-life of seconds, possibly due to the existence of circulating endonucleases [6]. It is noteworthy that sample treatments may affect the detection of non-coding RNAs. When comparing the potential of non-coding RNAs with traditional/emerging protein biomarkers for the early detection of MI, cardiac circRNAs were largely undetectable in heparinase treated samples, but microRNAs showed promise although suffered from lower detection sensitivity as compared with protein biomarkers [90].

Nevertheless, several studies have attempted to evaluate the potential of circular RNAs as biomarkers for heart disease (Table 1). In a large cohort study with 642 participants, a circulating circRNA, *MICRA*, was found to have a lower expression level in MI patents’ group and showed promise in predicting left ventricular function after MI [91]. Another large cohort study with 769 participants identified *hsa_circRNA_025016* as a potential biomarker for predicting postoperative AF after cardiac surgery [92].

There were several circRNAs identified as potential biomarkers for the coronary artery disease (CAD). *Hsa_circ_0124644* as a diagnostic biomarker for CAD was originally identified in an microarray profiling of blood samples from 12 patients of CAD and 12 control individuals, and was further tested with a larger cohort (115 control and 137 CAD patients) [93]. *Hsa_circ_0001879* and *Hsa_circ_0004104* were identified as upregulated in a profiling study using peripheral blood mononuclear cells from 24 CAD patients and 7 control, and were further validated in a larger cohort [94]. The combined use of these two circRNAs and CAD risk factors improved diagnostic performance. Moreover, the overexpression of *hsa_circ_0004104* in THP-1-derived macrophages regulated the expression of certain atherosclerosis-related genes, thus supporting its role in the pathogenesis of CAD. A highly abundant circRNA *hsa_circ_0001445* exhibited remarkable stability, as indicated by unperturbed detection even after prolonged room temperature storage or repetitive free/thaw cycles. Of note, *hsa_circ_0001445* has been evaluated in a real-world clinical practice setting as a biomarker for CAD [95]. The level of *hsa_circ_0001445* was inversely proportional to the extent of coronary atherosclerosis. Although not suggested as an independent biomarker for CAD, the addition of *hsa_circ_0001445* to a clinical model that includes other CAD risk factors significantly improved classification of patients, thus supporting its role as complementing existing diagnosis tools of CAD.

These studies support that certain circRNAs can be employed as independent biomarkers, while others can be added to the existing diagnostic toolset to further optimize it. Interestingly, several recent studies demonstrated that there are abundant circular RNAs in exosomes and extracellular vesicles [96,97,98,99,100,101,102,103,104]. In particular, circular RNA profiles in extracellular vesicles in murine hearts can be altered post ischemia/reperfusion injury [105]. Thus, future investigation on circular RNAs enriched in exosomes and extracellular vesicles may lead to the identification of more candidates for specific disease biomarkers. As more knowledge gained in the origin, function and working mechanism of circRNAs, their value as biomarkers will become clearer.

## 8. Perspectives

The aforementioned studies provide ample evidence that certain circRNAs act as molecular regulators of cardiovascular disease. The functional roles of several circRNAs have been uncovered in the setting of HF, using experimental models including TAC, ISO or AngII induced hypertrophy, Dox-dependent dilation as well as MI models. Almost all these models are reported to develop diastolic dysfunctions, and sometimes also exhibit systolic dysfunctions [73,106,107,108]. However, current studies largely focus on the functional effect of altered circRNA expression on systolic function. It would be informative to examine the roles of circRNAs in diastolic dysfunctions, because nearly half of the HF patients have a preserved ejection fraction [108]. Moreover, it is known that HF is associated with increased arrhythmia burden [109,110], which suggests that some of the differentially expressed circRNAs in the setting of HF may play a role in regulating heart rhythm.

Studies so far found that functional circRNAs mainly serve as microRNA or protein sponges to modulate heart diseases. While this line of research can provide a pool of candidates for therapeutic translation, only a small portion of circRNAs appears to serve as sponges [36,111]. Currently, knowledge of circular RNAs functioning in other modes is limited. Specifically, the relevance of ciRNAs and EIciRNAs to heart disease is unclear. Studies on the ecircRNA populations from the Titin gene have revealed that the lariat RNA pathway is specifically regulated by RBM20 in hearts [27,34]. RBM20, as a splicing regulator, also regulates circRNA production from a handful of other genes in hearts, whose involvement in heart disease remains to be tested [34]. On the other hand, the molecular basis for circRNAs to interact with various protein factors—specifically and efficiently—remains unexplored. Studies on other circular non-coding RNAs, such as viroids, have shown that RNA 3-dimensional motifs are particularly critical for RNA-protein, RNA-RNA and RNA-ligand interactions [112]. A comprehensive understanding of the structure-function relationships of some circRNAs will benefit future mechanistic studies and applications.

**Figure 3:**
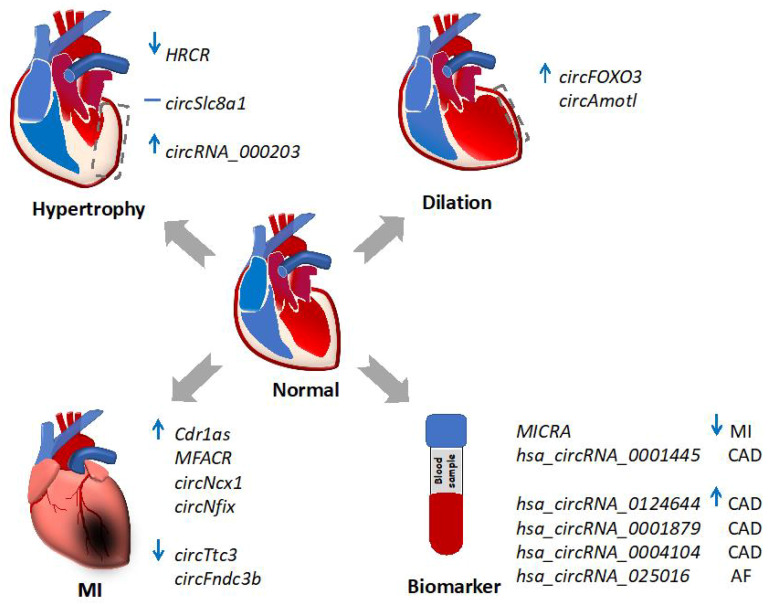
circRNAs as potential targets for the development of theranostics for heart disease. Gray boxes with dashed lines indicate pathological remodeling in cardiac hypertrophy and dilation. Blue arrows depict gene expression changes, with upwards showing elevation and downwards showing repression. MI, myocardial infarction. CAD, coronary artery disease. AF, atrial fibrillation.

Novel knowledge of circRNAs’ regulation of the cardiovascular system has demonstrated their great potential in the development of treatment for heart disease (Figure 3). The group of circRNAs with known functions in heart disease (e.g., microRNA or protein sponges) (Table 2) may serve as potential therapeutic targets to modulate disease progression. Additionally, research on the expression atlas of circRNAs at different stages of heart disease may help identify effective biomarkers for diagnosis and prognosis. Of note, the versatile functional modes of circRNAs also support their role as unique therapeutic molecules. For instance, circRNAs may potentially be used to deliver gene products to living cells. Engineered artificial circRNAs with an IRES sequence upstream of an open reading frame has been shown effectively expressing exogenous proteins in cells [113]. In addition, engineered circRNAs can be used to sponge microRNAs with known deleterious functions in cardiovascular disease, even though there is no such natural sponge in cells. This strategy was successfully tested in cancer research [114]. The advent of a deeper understanding of the functional mechanism of circRNA in diseased hearts will help to fulfill their potential for diagnostic and therapeutic applications.

## Figures and Tables

**Table 1:** List of potential circRNA biomarkers in heart disease. MI, myocardial infarction. AF, atrial fibrillation. CAD, coronary artery disease.

Disease	circRNA	Gene Synonym	Expression Profile	Potential Application	Ref
MI	*MICRA*	ZNF609	Repressed	predicting left ventricular function after MI	[91]
AF	*hsa_circRNA_025016*	CACNA1C	Elevated	predicting postoperative AF after cardiac surgery	[92]
CAD	*hsa_circRNA_0124644*	ROBO2	Elevated	diagnostic biomarker of CAD	[93]
	*hsa_circRNA_0001879*	NIPSNAP3A	Elevated		[94]
	*hsa_circRNA_0004104*	SPARC	Elevated		[94]
	*hsa_circRNA_0001445*	SMARCA5	Repressed		[95]

**Table 2:** List of functional circRNAs associated with heart failure (HF). All the studies cited in the table provided both in vitro and in vivo evidence. MI, myocardial infarction.

Disease Model	Circular RNA	Gene Synonym	Model/ Species	Expression in Disease	Implication in HF	Mechanism	Ref
Hypertrophy	*HRCR*	Pwwp2	mouse	repressed	overexpresssion alleviates hypertrophy	sponges miR-223 to regulate ARC	[67]
	*circSlc8a1*	Slc8a1	mouse	unaltered	downregulation attenuates hypertrophy	Sponges miR-133	[69]
	*circRNA_* *000203*	Myo9a	mouse	elevated	overexpresssion aggravates hypertrophy	sponges miR-26b-5p and miR-140-3p to regulate Gata4	[72]
Dilation	*circ* *FOXO3*	Foxo3	mouse human	elevated in aged hearts	overexpresssion aggravates dilation	interacts with ID-1, E2F1, FAK and H1F1	[46]
	*circAmotl*	Amotl1	mouse, human	higher level in neonatal hearts	overexpresssion alleviates dilation	interacts with AKT and PDK1	[47]
MI	*Cdr1as*	Cdr1	mouse	elevated	overexpresssion aggravates MI	sponges miR-7a	[75]
	*MFACR*	Smyd4	mouse	elevated	downregulation attenuates MI	sponges miR-652-3p to regulate MTP18	[76]
	*circNcx1*	Slc8a1	mouse	elevated	downregulation attenuates I/R	Sponges miR-133 to regulate CDIP1	[71]
	*circTtc3*	Ttc3	rat	repressed	downregulation aggravates MI	sponges miR-15b-5p to regulate Arl2	[77]
	*circNfix*	Nfix	mouse, rat, human	elevated	downregulation alleviates MI	interacts with YBX1 and NEDD4L to degrade YBX1; sponges miR-214 to regulate Gsk3β signaling	[78]
	*circFndc3b*	Fndc3b	mouse, human	repressed	overexpression alleviates MI	interacts with FUS to regulate VEGF-A	[79]
